# Acoustic Emission Signal Entropy as a Means to Estimate Loads in Fiber Reinforced Polymer Rods

**DOI:** 10.3390/s21041089

**Published:** 2021-02-05

**Authors:** Mohammadhadi Shateri, Maha Ghaib, Dagmar Svecova, Douglas Thomson

**Affiliations:** 1 Department of Electrical & Computer Engineering, McGill University, Montreal, QC H3A 2K6, Canada; mohammadhadi.shateri@mail.mcgill.ca; 2 Department of Civil Engineering, University of Manitoba, Winnipeg, MB R3T 2N2, Canada; ghaibm@myumanitoba.ca (M.G.); Dagmar.Svecova@umanitoba.ca (D.S.); 3 Department of Electrical and Computer Engineering, University of Manitoba, Winnipeg, MB R3T 2N2, Canada

**Keywords:** acoustic emission signal, fiber reinforced polymer rod, service load, Shannon entropy, Chebyshev’s inequality

## Abstract

Fibre reinforced polymer (FRP) rods are widely used as corrosion-resistant reinforcing in civil structures. However, developing a method to determine the loads on in-service FRP rods remains a challenge. In this study, the entropy of acoustic emission (AE) emanating from FRP rods is used to estimate the applied loads. As loads increased, the fraction of AE hits with higher entropy also increased. High entropy AE hits are defined using the one-sided Chebyshev’s inequality with parameter *k* = 2 where the histogram of AE entropy up to 10–15% of ultimate load was used as a baseline. According to the one-sided Chebyshev’s inequality, when more than 20% (*k* = 2) of AE hits that fall further than two standard deviations away from the mean are classified as high entropy events, a new distribution of high entropy AE hits is assumed to exist. We have found that the fraction of high AE hits. In glass FRP and carbon FRP rods, a high entropy AE hit fraction of 20% was exceeded at approximately 40% and 50% of the ultimate load, respectively. This work demonstrates that monitoring high entropy AE hits may provide a useful means to estimate the loads on FRP rods.

## 1. Introduction

### 1.1. Motivation

**Figure 1:**
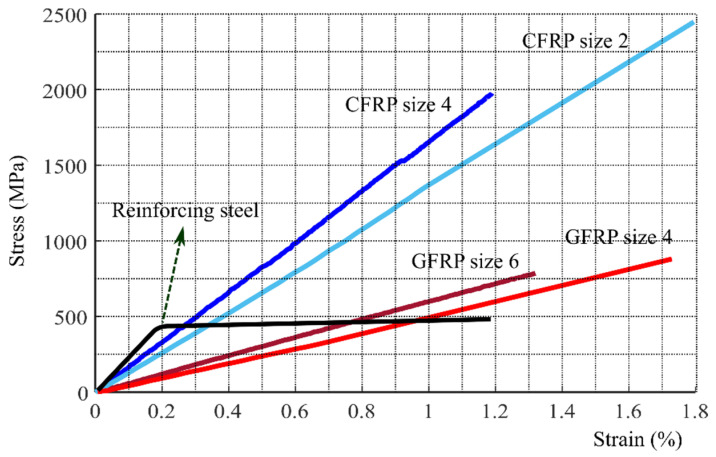
Stress-strain characteristics of various types of reinforcement.

Fiber reinforced polymer (FRP) rods have been widely used for reinforcing in civil structures including concrete structures and steel structures [1,2]. High strength-to-weight ratio and high resistance to the corrosion make FRP rods a good replacement for steel reinforcing rods [2]. According to [3] up to 2012, FRP rods have been used in the construction of 190 installations in Canada including bridge decks, parapets, barriers, sidewalks and in the U.S. they have been used in more than 50 bridge decks. FRP rods can be used as reinforcing as long as the allowed service load is not exceeded. To prevent creep failure, standards for the use of FRP bars such as ACI440.1R and CAN/CSA-S806-12 recommend maximum stress levels under service loads should not exceed 20–25% and 55–65% of the ultimate strength for glass FRP (GFRP) and carbon FRP (CFRP). One of the main drawbacks of the FRP rods compare to the steel reinforcing rods is their lack of ductility (see Figure 1). Therefore, it is a matter of great importance to develop a technique for determining the applied loads on FRP rods. Ideally the technique would be non-destructive and not require a baseline measurement, as a baseline measurement will not be possible for structures constructed years or even decades in the past.

### 1.2. Related Work

**Figure 2:**
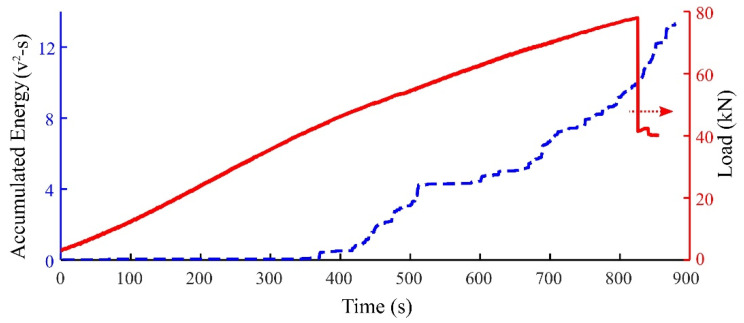
Temporal evolution of AE hits energy against applied load for a CFRP size 2 specimen.

Studies have shown that acoustic emission (AE) signal analysis is a promising non-destructive technique for studying failure in concrete, e.g., within non-extensive statistical mechanics framework [4,5,6], damage localization in composite structures [7,8] and for damage monitoring in FRP materials [9,10]. When damage occurs in FRP materials, the sudden change in stress produces AE signal. Figure 2 represents how AE energy is accumulated by applying mechanical load. Many studies have used time-based features extracted from AE hits, i.e., peak amplitude, duration, energy and so on to characterize different damage mechanisms in FRP materials [11,12]. Some studies used a group of these features, combined with pattern recognition techniques to discriminate the AE hits into clusters and then detect damage mechanisms based on the clusters [13,14,15].

Many approaches use measures that are dependent on the magnitude of signals, which is problematic as the attenuation is high and the distance between source and sensor is highly variable.

Ni et al. [16] showed that the sensors location can have a dramatic effect on the amplitude attenuation and hence the time descriptors of AE hits can be changed while the normalized frequency spectrum is unchanged. They concluded that fast Fourier transform (FFT) and time-frequency method of wavelet transform (WT) are promising signal analysis tools for investigation of microfailure modes and microfracture mechanisms in composite materials. AE hits collected from FRP materials were investigated based on the frequency content in [17,18,19] using the Fourier transform (FT). These studies suggested that each damage mechanism can be associated with a different frequency band. In some studies [20,21] based on the fact that WT provides better frequency resolution compare to the FT, the WT was used to discriminate the AE hits. Using the WT each AE hit was decomposed into different frequency levels and the energy of each level was used for identification of damage mechanisms. Even though there have been many studies for identification of damage mechanisms in FRP materials, none of them have explored a methodology to investigate when the in-service loading of FRP rods. In a study conducted by Unnthorsson et al. [22] the randomness of AE hits investigated using entropy was used for prediction of the early failure in FRP materials. In that study four different entropies were defined in time and in frequency. Their results showed that the evolution of average entropy is almost unchanged from 20% to 95% of the FRP’s lifetime. Therefore, they concluded that the average entropy cannot be used for early failure prediction. However, that work analyzed the entire time series of the signal.

### 1.3. Contributions

In the present work, we adopt a frequency-based approach and use the Shannon entropy of the AE hits to estimate the loads in FRP rods. Unlike the method presented in [22] where the entire time series of the signal was analyzed, we isolate the individual AE hits (hits) and analyze the entropy of the events rather than the entire signal. The benefit is twofold. First, this provide an indicators of high and low entropy AE hits since when the energy of an AE hit is distributed in a few frequencies, the frequency spectrum is less random, and the entropy is lower. On the other hand, in a more random signal the energy is more evenly distributed in the frequency spectrum and the entropy is higher. Second, analyzing isolated AE hits dramatically reduces the effect of background noise on the calculated entropy. It should be noted that in the literature, in studies such as [23,24,25] the total energy content of an AE hit is used to study damage in composites while in the present study, the presented method is based on how energy is distributed in different frequencies. In other words, unlike the previous studies that are based on an increase in AE hits energy, our method is based on uncertainty in energy distribution over frequency. Using entropy has the significant advantage in that it is self-normalizing and does not rely upon a predefined absolute reference. In this work we have found significant variation in the energy per hit and in integrated hit energy totals. Due to this sample to sample variation, the load could not reliably be predicted using in hit energy or integrated hit energy. In this work, entropies are calculated in two ways according to the frequency spectrum of the AE signal found by FT and WT. These entropies measure the randomness of the energy distribution of AE hits over different frequencies. The idea of using entropy in frequency for damage detection is based on the hypothesis that different frequency bands can be associated with different damage mechanisms [26]. In more highly damaging events, it is expected that multiple damage mechanisms will be involved and hence the energy of the AE hit will be distributed across more frequencies. Consequently, the AE signals emitted from more severely damaging events in FRP would be expected to have high entropy. The one-sided Chebyshev’s inequality with parameter k [27] is used to define the high entropy in the histogram of AE entropy. According to this inequality whenever the number of AE hits with high entropy is greater than 1/(1 + *k*^2^) × 100% a new distribution in the right hand tail of AE entropy histogram is said to exist. In this work, the existence of this high entropy AE hits distribution is used as an indicator of the in-service load on FRP rods.

## 2. Materials and Methods

**Figure 3:**
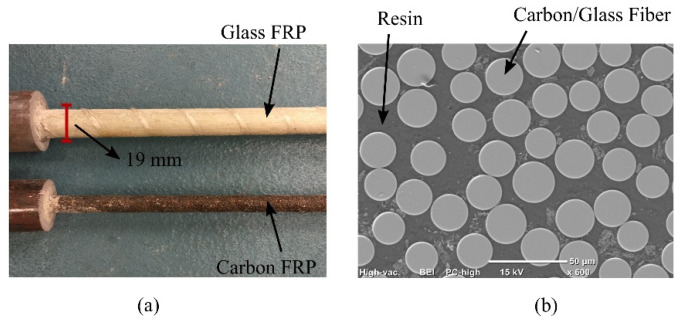
(**a**) Morphology of fiber reinforced polymer bars (**b**) Scanning electron microscope (SEM) image of fiber reinforced polymer bar.

FRP bars are a combination of fibers embedded in resin matrix (polymer) which protects the fibers and helps in transferring of stress between individual fibers. Fibers are the main load-bearing components in FRP bars. Compared to steel, the resin matrix is very resistant to corrosion but has lower mechanical strength. On the other hand, fibers exhibit high strength and stiffness. In this study, FRP rods were bonded into cylindrical steel anchors. The assemblies were mounted into load frames and ramping tensile loads were applied until the rods failed. Figure 3 represents samples of FRP bars used in this experiment.

In this study, 9 FRP rods from two different types of FRP materials including glass FRP (GFRP) and carbon FRP (CFRP) are used. The specimens are manufactured according to the CSA S806-06 (2006) Annex B standard. Table 1 lists the tested FRP rods.

Tensile test on the prepared FRP rods is carried out using an Instron 300DX universal testing machine and a 30 kip Baldwin universal testing machine under a ramping load. Table 2 represents the details of loading.

**Figure 4:**
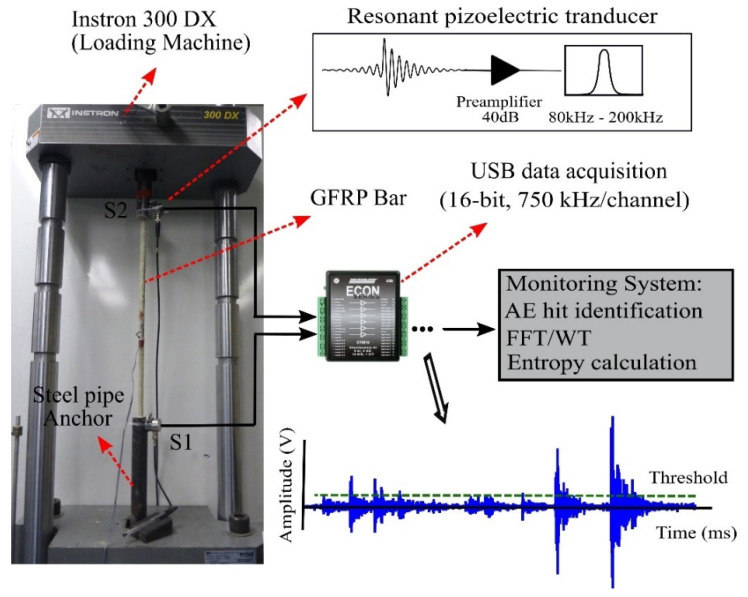
A schematic of the FRP tensile test.

A resonant ARI5I-AST integral preamplifier piezoelectric sensor of operating frequency 80 kHz to 200 kHz that has 40 dB low noise preamplifier built in was used as an acoustic signal sensor. The signals were digitized and logged using a data acquisition system (DT9816-S, 16-bit, 750 kHz per channel). A Proceq couplant gel (Part No. 71010031) was injected between the face of the sensor and the surface of the steel anchor to facilitate the transfer of AE waves to the sensor. A pencil-lead break test is performed for calibration of sensor [28] and to set appropriate values for the parameters of AE hit detection algorithm. It should be noted that the RMS hit detection method presented in [10] is used. For more details of the RMS approach and the parameters associated to AE hits identification (such as threshold value), the readers are referred to [13]. Figure 4 is a schematic diagram of the FRP tensile test set up. The logged signals were analyzed to first find each hit, and then each hit signal was analyzed to determine the entropy. The Shannon entropy or information entropy that was proposed by Shannon [29] is a measure of randomness or uncertainty of a random variable. Let X be a random variable with probability mass function (PMF) Pr(*x*), where $x\in \left\{x_{1},x_{2},\;\ldots ,\;x_{M}\right\}$ is the range of the random variable *X* and $\sum_{i=1}^{M}\mathrm{Pr}\left(x=x_{i}\right)=1$. The Shannon entropy for the random variable *X* is defined as follows:

(1) $$H\left(X\right)=\;E\left[\mathrm{Pr}\left(x\right)\right]=\;-\overset{M}{\underset{i=1}{\sum }}\mathrm{Pr}\left(x=x_{i}\right)\log\left(\mathrm{Pr}\left(x=x_{i}\right)\right)\;$$

where expectation E[.] is with respect to Pr(*x*). For each AE hit detected using RMS technique during the tensile test of FRP rods, the frequency content can be considered as a random variable where its probability function is defined as the frequency spectrum normalized by the total magnitude of spectrum [22].

**Figure 5:**
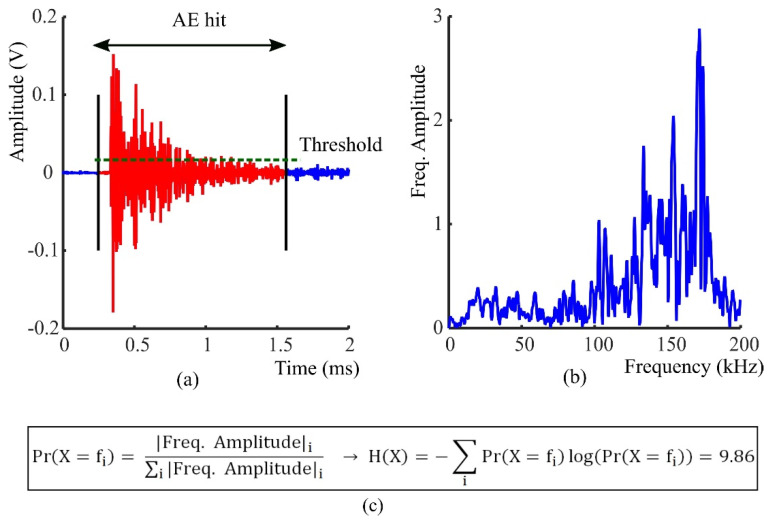
AE entropy calculation (**a**) Detected AE hit using RMS algorithm [8] (**b**) Frequency spectrum of AE hit (**c**) Applying the Shannon entropy formula to the normalized spectrum. In the example shown the entropy is 9.86.

The Shannon entropy in (1) is used to find the entropy of acoustic emission signals (See Figure 5). In this sense the acoustic emission entropy represents a measure of uncertainty about the energy distribution in different frequencies. In other words, if the energy of acoustic signal is distributed in a few frequencies the entropy is small, on the other hand a large entropy is expected when the energy is more evenly distributed in frequency. In this study the entropy of acoustic signals is calculated using Fourier transform and Wavelet transforms, as explained below.

For a sequence of N samples of a signal $\left\{x_{1},x_{2},\;\ldots ,{\;x}_{N}\right\}$ the discrete Fourier transform (DFT) is defined as follows [30]:

(2) $$X_{k}=\overset{N-1}{\underset{n=0}{\sum }}x_{n}e^{-\frac{j2\pi \mathrm{kn}}{N}},\;\;\;\;\;\;\;\;k=0,\;1,\;\ldots ,\;N-1$$

where $\left\{X_{k}\right\}\;,k=0,\;1,\;\ldots ,\;N-1$ are the DFT coefficients or frequency samples of signal $x_{n}$. According to the Shannon entropy defined in (1), in order to compute the entropy a probability function is defined in frequency. For a pure real signal $x_{n}$ the DFT is semmetric i.e., $X_{N-k}=\;X_{k}{}^{*},\;k=0,\;1,\;\ldots ,\;N-1$ where * here means complex conjugation. Therefore, using the first half of DFT coefficients (positive frequencies) the PMF in the frequency domain can be defined as follows [26]:

(3) $$\mathrm{Pr}\left(X=X_{i}\right)=\;\frac{\left|X_{i}\right|}{\sum_{j=0}^{\frac{N}{2}-1}\left|X_{j}\right|}\;\;\;\;\;\;\;\;\;\;\;\;\;\;i=0,\;1,\;\ldots \;,\;\frac{N}{2}-1\;\;\;\;\;\;\;\;\;\;$$

where $\left|X_{i}\right|$ is the magnitude of signal in the *i*-th frequency. It should be noted that by considering $N=\;2^{l}$ where *l* is an integer, the DFT (2) can be computed using very efficient fast Fourier transform (FFT) algorithms [30]. In this study FFT algorithms are used to calculate the frequency spectrum of acoustic signals. In this work the number of FFT points is chosen to be the power of two closest to the length of AE hits. For those AE hits that have less than the required number of points, zero-padding is used before applying DFT, which results in a frequency interpolation.

The wavelet transform was introduced in the early 1980s [31] and has been used as a useful tool for analyzing transient signals [32]. The continuous wavelet transform (CWT) of a signal *f*(*t*) is defined as follows:

(4) $$C\left(a,b\right)=\;\frac{1}{\sqrt{a}}\int f\left(t\right)\psi^{*}\left(\frac{t-b}{a}\right)dt$$

where ψ(t) is the mother wavelet, a refers to the scale parameter (frequency) and b represents the shifting parameter (time). By defining the scale parameter a as $2^{-j}$ and the shifting parameter equal to $k2^{-j}$ the discrete wavelet transform can be computed as follows [31]:

(5) $$C_{j,k}=2^{\frac{j}{2}}\int f\left(t\right)\psi^{*}\left(2^{j}t-k\right)dt$$

where the resolution level *j* and the sample time *k* are integers. The number of levels in wavelet transform should be less than the log two of the length of AE hits. In some studies for higher resolution frequency analysis, non-integer values are selected for the resolution level *j* [32]. The wavelet transform can be done using different mother wavelets. In this study because of the similarity with acoustic emission signals, the Daubechies wavelets with 10 vanishing moments are used [33]. Using the wavelet coefficients (3.5) the energy of the signal in each resolution level can be defined as follows:

(6) $$E_{j}=\;\underset{k}{\sum }{\left|C_{j,k}\right|}^{2}$$

By defining the total energy as $E_{t}=\;\sum_{j}\sum_{k}{\left|C_{j,k}\right|}^{2}$ the probability function can be represented as the relative wavelet energy [33]:

(7) $$\mathrm{Pr}\left(x_{j}\right)=\;\frac{E_{j}}{E_{t}}$$

Therefore, by substituting (7) in the Shannon entropy (1) the wavelet entropy can be defined.

Assume X is a random variable with expected value µ and variance $\sigma^{2}$. The Chebyshev’s inequality proposed for the first time in [34] can be defined for X as follows:

(8) $$\mathrm{Pr}\left(\left|x-µ\right|\geq k\sigma \right)\;\leq \frac{1}{k^{2}}$$

where *k* ˃ 1 is integer. In other words, for any random variable x, with a chance of more than $1-\frac{1}{k^{2}}$, the values are within k standard deviation of the mean. Based on (4.1) appearing more than $\frac{1}{k^{2}}$ percent of samples outside the *k* standard deviation of the mean, means more than one distribution exists. There are different extensions for the Chebyshev’s inequality. The one-sided version of Chebyshev’s inequality can be stated as follows:

(9) $$\mathrm{Pr}\left(x-µ\geq k\sigma \right)\;\leq \frac{1}{1+k^{2}}$$

This version has been recognized as Cantelli’s inequality that is a generalized Chebyshev’s inequality for one tail of the distribution [27]. In this study the one-sided Chebyshev’s inequality will be applied to the entropy distribution of acoustic emission event signals detected during the FRP tensile test to determine the load on FRP rods. The parameters µ and σ of the entropy histogram are estimated using the detected AE hit generated as the load is ramped up to 10–15 percent of the ultimate load to FRP rods. Up to 10–15 percent of the ultimate load, the entropies have almost a normal distribution except for a small number of events that can be classified as outliers from a normal distribution. Up to 10–15 percent of the ultimate load the damage appears to be from one dominant type and the frequency spectrum of AE hit is concentrated in a few frequencies corresponding to the type of damage [26]. Therefore the AE hits with low entropy are detected that make the left tail of distribution longer. When more damage types are present the frequency spectrum of detected AE hits are spread over more frequency bands, leading to higher entropy AE hits. This increase in the number of high entropy AE hits leads to a new distribution in the right tail of AE entropy histogram. Using one-sided Chebyshev’s inequality, derived from the initial AE hits (up to 10–15 percent of ultimate load) the existence of a new distribution in the right tail can be detected and is used to provide and indicator of the load on the FRP rods.

One issue concern with using Chebyshev’s inequality is finding the mean and standard deviation. Since FRP rods can be damaged in the early stages of loading, the entropy of AE hits sourced from this initial damage can affect the calculation of mean and standard deviation. A robust estimation of µ and σ can be done using the median and median absolute deviation (MAD). For a data set $x_{1},x_{2},\;\ldots ,{\;x}_{N}$ the MAD is computed as follows:

(10) $$\mathrm{MAD}={\mathrm{median}}_{i}\left(\left|x_{i}-{\;\mathrm{median}}_{j}\left(x_{j}\right)\right|\right)$$

The mean of data set can be estimated using the median and if we assume a normal distribution for the data set the standard deviation is estimated as follows [35]:

(11) σ≈1.4826×MAD 

## 3. Results and Discussion

**Figure 6:**
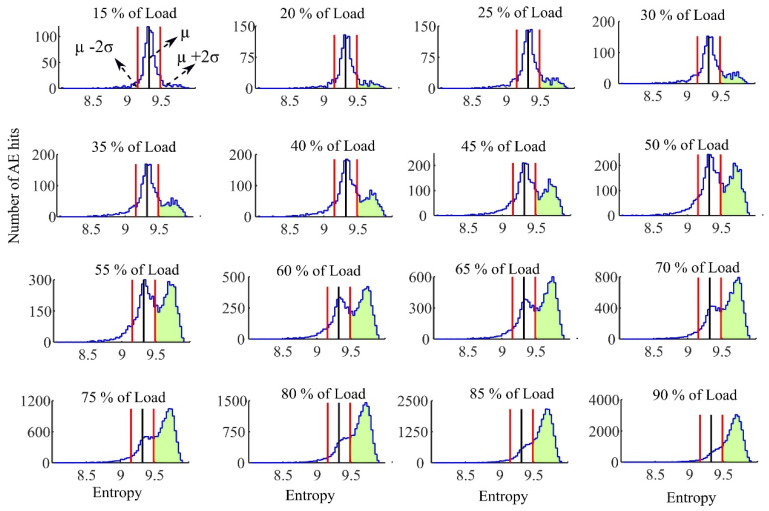
Histogram of the entropy using Fourier transform in different percent of ultimate load.

**Figure 7:**
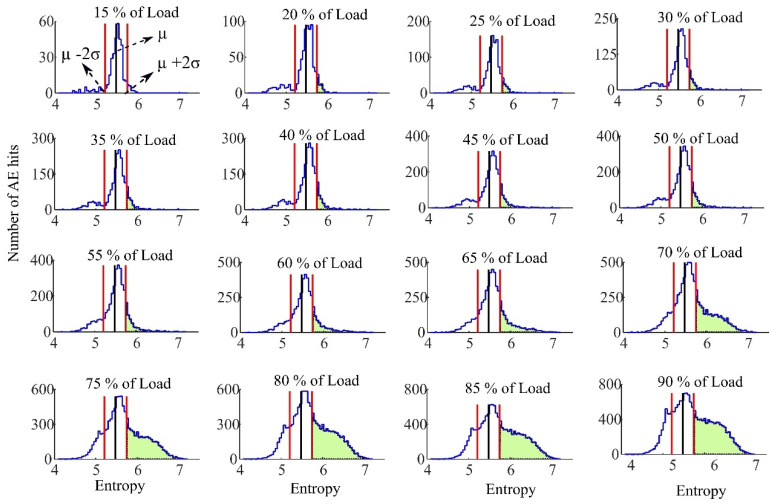
Histogram of the entropy using wavelet transform in different percent of ultimate load.

The tensile test was carried out under a ramping load. According to the frequency range of AE sensor a sampling frequency of 400 kHz was selected for the data acquisition system. A threshold value equivalent to ten times of the noise level was used to minimize false event detection. Only AE hits that crossed this threshold were detected. The acoustic emission entropy for each detected event was defined by applying a 2048-points discrete Fourier transform (DFT) and a wavelet transform with a scale parameter varying from 1 to 16 by 0.1 step. Figure 6 and Figure 7 represent the histogram of Fourier transform entropy and Wavelet transform entropy in different percent of ultimate load respectively. The histogram of AE entropy at 10–15 percent of ultimate load was used for extracting the parameters for the Chebyshev’s inequality.

The middle vertical line in each histogram is the mean µ of the histogram for AE hits up to 10–15 percent of the ultimate load. The region between the left- and right-hand vertical lines is that which is within two standard deviations from this mean. These figures show that during the early stages of loading the histogram of the entropy has a shape similar to a normal distribution. Applying increasing load to the FRP rods results in a left tail of distribution longer that is related to smaller entropy events. These small entropies mean the frequency spectrum of detected AE hit has a reduced frequency range and can be due to a specific type of damage being dominant at lower loads of the test. When other types of damage increase substantially, the frequency spectrum of AE hits contains a broader frequency content, due to the diverse damage types [26]. The broader frequency content increases the AE entropy for each event. This leads to the appearance of AE hits with high entropy (shaded regions). The right tail of AE entropy histogram is longer and a new distribution of high AE entropy events formed in the right tail. It can be seen that since both left and right tails of the entropy distribution are increased, the average entropy would not change significantly. However, the right tail that is due to the more damaging events of FRP rods can be used to determine the load. The one-sided Chebyshev’s inequality is applied to detect this new distribution in the right tail.

**Figure 8:**
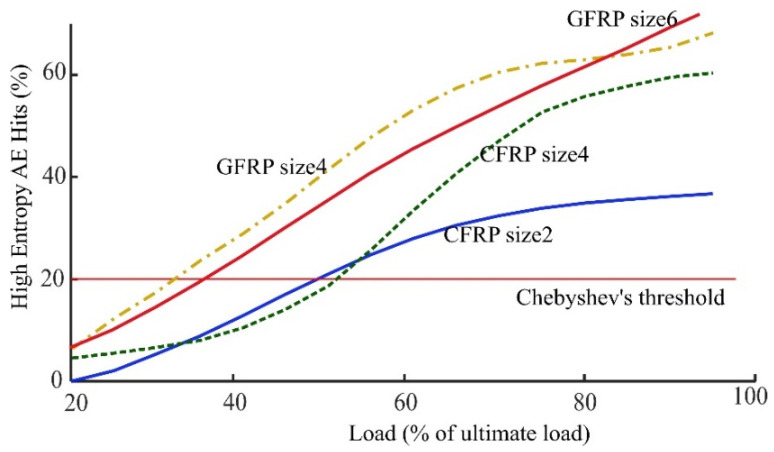
Evolution of the number of AE hit with high entropy using Fourier transform in different percent of ultimate load for different FRP rods.

According to the one-sided Chebyshev’s inequality and by selecting *k* = 2 those AE hits that have entropy more than two standard deviation from the mean are considered as the high entropy AE hits. Whenever the fraction of these high entropy AE hits passes the $\frac{1}{1+k^{2}}$ (20% for *k* = 2) a new distribution in the right tail is assumed to exist. As it was mentioned this new distribution of high entropy AE hits is a result of noticeably different type of damages in FRP rod. It is of particular note that the emergence of a new distribution high AE entropy events is correlated with the loads on the FRP rods reaching the recommended service load. Figure 8 exhibits the percent of amount of high entropy AE hits detected using Chebyshev’s inequality with *k* = 2 for Fourier transform entropy versus different percent of ultimate load (for the Wavelet transform entropy the figure would be very similar—not presented here-). In addition, Table 3 lists the results of exceeding the Chebyshev’s threshold for all the FRP bars specimens.

From this table it can be seen that the evolution curve of high entropy AE hits surpasses the Chebyshev’s threshold around 50% of ultimate load for the CFRP rods and around 40% of ultimate load for the GFRP rods. It means high entropy AE hits within the FRP rods, that are the result of simultaneous independent damage mechanisms in the spectrum of AE hits, make a new distribution at this point. According to the Canadian standard association (CSA) design and construction of building component with FRP (S806-12), at serviceability limit state the load on the FRP bars shouldn’t exceed 25% and 65% of ultimate load for GFRP and CFRP respectively. Therefore, the emergence of this new distribution of high entropy AE hits is correlated with reaching the recommended service loads. In other words, when the maximum service load is exceeded, the number of high entropy AE hits surpasses the Chebyshev’s threshold and make a new distribution of high entropy AE hits. In addition, as the load increases the fraction of high entropy events also increases. The relationship between the applied load and the fraction of high entropy events is monotonic in three of the four cases over the full load range. In all cases the relationship between the applied load and the fraction of high entropy events is monotonic up to 70% of the ultimate load range.

## 4. Conclusions

The purpose of this study was to develop a non-destructive means to determine the load exerted on FRP rods. To this end, the AE signals collected during an FRP tensile test under ramping loads were used, where AE hits were identified using RMS hit detection algorithm. For each detected AE hit, the frequency spectrum was found using the FT and WT and then the Shannon entropy was calculated accordingly as a measure of the randomness of AE hits energy distribution over frequency. The results of testing nine different FRP rods showed the following important points:

- When the load was less than 10–15% of the ultimate load, the AE entropy had approximately a normal distribution.
- When loads exceeded 10–15% of the ultimate load, AE hits with higher entropies were observed where the one-sided Chebyshev’s inequality (generated with the histogram of AE entropy at 10–15% of ultimate load) showed to be useful in detecting the emergence of a new entropy distribution.
- The emergence of a new distribution with high AE entropy events was correlated with loads exceeding the recommended service load limits for FRP rods. This occurred between 32–42% and 45–65% of ultimate load for GFRP and CFRP, respectively. These values correlated with the recommended maximum stress levels of 20–25% and 55–65% of the ultimate strength provided by standard codes for FRP bars such as ACI440.1R and CAN/CSA-S806-12.

Therefore, this work suggests that monitoring high entropy AE hits should be explored as a means for monitoring loads on in-service FRP rods. This techniques should also be tested for fatigue damage that is also expected for in-service FRP reinforcing. This technique could have important application in monitoring FRP rods in prestressing applications and in perhaps in monitoring the in-service behavior of FRP rods.

## Figures and Tables

**Table 1:** List of FRP specimens.

Bar Type	Diameter (mm)	Gauge Length (mm)	Surface Coating
CFRP size 2	6	240	Sand coated
CFRP size 4	13	520	Sand coated
GFRP size 4	13	520	Undulation and sand coated
GFRP size 6	19	760	Undulation and sand coated

**Table 2:** FRP tensile test load information. The displacement rate is calculated based on the CAN/CSA-S806-12 code for FRP bars.

Bar Type	No. Specimens	Displacement Rate $\left(\frac{mm}{min}\right)$	Ultimate Load (kN)
CFRP size 2	3	0.8	78
CFRP size 4	2	1.8	250
GFRP size 4	2	4	117
GFRP size 6	2	6	240

**Table 3:** List of the results of exceeding the Chebyshev’s threshold for all the FRP bar specimens.

FRP Bar Type		Chebyshev’s t = Threshold Crossing (Percent of Ultimate Load %)	
		FFT Entropy	WT Entropy
**CFRP size 2**	Specimen1	65	65
	Specimen2	45	45
	Specimen3	55	55
	**Average**	**53.33**	
**CFRP size 2**	Specimen1	57	48
	Specimen2	50	49
	**Average**	**51**	
**GFRP size 4**	Specimen1	34	32
	Specimen2	37	35
	**Average**	**34.5**	
**GFRP size 6**	Specimen1	42	32
	Specimen2	38	36
	**Average**	**37**	

## Data Availability

Please contact D.J. Thomson.
